# Mr. Wawn, on Burns

**Published:** 1801-01

**Authors:** Chas. Newby Wawn

**Affiliations:** Newcastle upon Tyne


### *To the Editors of the Medical and Phyfical Journal.*

GENTLEMEN,

AT this time, when medical men are fo much at variance concerning the treatment of burns and fcalds, it behoves every one one to contribute to the general ſtock of facts, upon which, rather than upon any pre-conceived theory, this new doctrine muſt be eſtabliſhed.—If you deem the following caſe ſufficiently intereſting to merit a place in your valuable Journal, by ſo doing you will much oblige,

GENTLEMEN,

*Newcaſtle upon Tyne,* Your humble ſervant,

*Dec.* 3, 1800. CHAS. NEWBY WAWN.

---

G. T. aged 14, ſtanding near a fire, with a caniſter which contained a conſiderable quantity of gunpowder in his hand, wantonly threw a few grains into the fire; the ſparks from which, communicating with the uncovered caniſter, produced an exploſion ſo violent as to burſt open the windows and door of the room in which he was. His right hand and the whole of his face were ſeverely burnt, the cuticle wholly abraded, and veſications formed; the eye-laſhes, eye-brows and hair on the forehead were entirely conſumed; he complained of violent pain, and ſhivered inceſſantly; pulſe 120.

On firſt ſeeing him, which was about twenty minutes after the accident, gts. xxx of laudanum were immediately adminiſtered, the ſpt. terebinth. was liberally applied to the whole of the inflamed ſurface, and above that, plaſters ſpread with Mr. Kentiſh's digeſtive ointment (Ung. reſin. flav. reduced to a thin conſiſtence with ſpt. teberinth). He was ordered to be put to bed. This occurred in the forenoon; I ſaw him again in the evening, when he declared himſelf quite eaſy and comfortable; had ſlept two or three hours, and during the afternoon had felt only at times a paroxyſm of pain, which was much leſs ſevere than at firſt; pulſe 112. A draught with gt. xx of laudanum was ordered to be taken at bed time. Second day, has paſſed a tolerably good night; the inflammation on the face appears leſs vivid. In removing the dreſſing from the inner part of the hand, the veſications broke, and a portion of the detached cuticle came off with it; from which, however, no unpleaſant ſymptom occurred; pulſe 105. Spt. terebinth. more ſparingly applied, other remedies continued. Third day, is materially better; has had a diſcharge from the angles of the eyes, which he has now opened for the firſt time; they appear ſlightly inflamed; purulent ſecretion beginning to take place on the hand, and partially on the face; pulſe 95. Draught omitted.

From this time ſtimulant applications were gradually withdrawn, and the parts were healed with cerat. lap. calam. which was completely effected in twelve days from the accident. The face is not in the ſmalleſt degree disfigured, nor is the ſlighteſt veſtige of the burn to be ſeen on the hand, of which he retains the perfect uſe.

